# Animal virus-host interactions mediated by non-coding RNAs

**DOI:** 10.3389/fcimb.2026.1881266

**Published:** 2026-07-07

**Authors:** Zelong Sun, Ye Wang, Zheng Zhou, Jianbo Liu

**Affiliations:** 1 Henan Key Laboratory of Precision Diagnosis of Respiratory Infectious Diseases, Zhengzhou Key Laboratory of Precision Diagnosis of Respiratory Infectious Diseases, The Second Affiliated Hospital of Zhengzhou University, Zhengzhou, Henan, China; 2 Center for Medical Experiment, The Second Clinical Medical School of Zhengzhou University, The Second Affiliated Hospital of Zhengzhou University, Zhengzhou, Henan, China

**Keywords:** animal virus, circRNAs, lncRNAs, miRNAs, ncRNAs networks, virus-host interactions

## Abstract

Non-coding RNAs (ncRNAs) have emerged as key regulators of animal virus-host interactions, such as lncRNAs, miRNAs and circRNAs providing profound insights into host-pathogen interactions and revealing significant potential as biomarkers and therapeutic targets. This review summarizes the interactions between viruses (mainly including animal viruses, zoonotic viruses, insect-vector virus systems and plant viruses) and hosts mediated by lncRNAs, miRNAs, circRNAs and other non-coding RNAs. We focus on the research related to zoonotic disease transmission, non-model animals, aquaculture and animal models, and highlight the critical molecular mechanisms of ncRNAs (including host-derived ncRNAs and viral ncRNAs) in regulating virus-host interactions and their therapeutic potential as antiviral drug targets. In addition, we discuss future research directions and prospects for animal virus-host interactions. Meanwhile, we highlight the application of ncRNA-based RNA vaccines in the animal disease prevention, with the ultimate goal of improving animal health and fostering harmonious coexistence between humans and animals.

## 1 Introduction

Viruses are the most diverse and abundant biological entities, which can infect all forms of life and parasitize in new host organisms, thereby triggering serious diseases (Wasik and Turner, 2013; Paez-Espino et al., 2016). The discovery of the first virus originated from prominent disease phenotypes. Subsequently, virology research has long focused on symptoms associated with apparent diseases, particularly concentrating on viruses directly relevant to humans in animals and plants (Kumar et al., 2017). In recent years, the emergence of new infectious diseases caused by animal viruses has significantly increased in global animal populations, posing substantial threats to both animal and human health (Daszak et al., 2000; Sarker et al., 2021). Numerous emerging viral pathogens-including Ebola virus, Nipah virus, Sin Nombre virus, Hendra virus, West Nile virus, Middle East Respiratory Syndrome (MERS), and various avian influenza subtypes-originate from animals and have caused major diseases affecting human health (Cunningham et al., 2017). Therefore, understanding the molecular mechanisms of virus-host interactions will provide will provide foundational knowledge for the development of antiviral vaccines and therapeutics.

Although most factors known to regulate the viral life cycle are proteins, an increasing number of ncRNAs have been proven to participate in these biological processes (Cai et al., 2022; Li et al., 2022). Up to now, with the rapid development of high-throughput sequencing technologies, an increasing number of ncRNAs regulating interactions between animal viruses and their hosts have been discovered and characterized (Bhargava et al., 2025; Wang Y. et al., 2025), such as long non-coding RNAs, microRNAs and circular RNAs (Xie et al., 2021; Liu et al., 2024; Wang et al., 2024). Ji et al. used human lncRNA microarrays to identify 896 differentially expressed lncRNAs in rabies virus (RABV)-infected and non-infected human neuroblastoma cells, providing deeper insights into the pathogenesis of RABV (Ji et al., 2019). Orf disease is a zoonotic disease. Through miRNA and RNA sequencing on goat skin fibroblasts infected with Orf virus (ORFV), Pang et al. found that a significantly upregulated miRNA, Cfa-let-7a, inhibits the expression of thrombospondin 1 (THBS1) by directly targeting the 3’untranslated region, and then plays an important role in cell apoptosis during ORFV infection (Pang et al., 2020). This study provides new insights into the interaction mechanism between ORFV and host cells. Vesicular stomatitis virus (VSV) is a negative-sense RNA virus that threatens the animal husbandry industry and currently lacks effective treatments. The latest research by Miao et al. identified 1682 host-derived differentially expressed circRNAs in Vero cells infected by VSV through high-throughput sequencing, top 10 of which were upregulated by VSV and enhanced VSV infection as well. Collectively, these findings demonstrate that both host- and virus-encoded circRNAs are induced following VSV infection and uncover a previously uncharacterized layer of virus-host crosstalk mediated by circRNAs, which may inform the development of novel antiviral strategies against VSV (Miao et al., 2025). Meanwhile, the COVID-19 pandemic has also brought new relevance to research in zoology and virus evolution, especially the interactions between animal viruses and their hosts (Chen et al., 2024). In summary, a growing number of studies have demonstrated the regulatory roles of ncRNAs in viruses and their hosts, highlighting the importance of protein-RNA and RNA-RNA interactions during infection (Wang et al., 2024; Zhang X. et al., 2024; Zoladek et al., 2024). Studies on the roles of ncRNAs in animal virus-host interaction is on the rise.

Therefore, in this review, we summarize recent research progress (especially over the past five years) on ncRNA-mediated animal virus-host interactions based on data retrieved from the NCBI database. The search keywords include animal viruses, ncRNAs, virus-host interaction and other related terms, and the covered viruses include livestock viruses, avian viruses, aquatic animal viruses, companion animal viruses, wildlife viruses, zoonotic viruses, viruses studied in human cell models and insect-vector-borne viruses. ncRNAs include host ncRNAs and viral ncRNAs. We mainly focus on the latest research regarding the functions and mechanisms of ncRNAs in animal virus-host interactions, including their potential as therapeutic targets, molecular biomarkers and other related roles. Moreover, we highlight that a special virus-host interaction system, the plant virus-insect vector-plant host system (such as rice stripe virus), represents cross-kingdom/vector-mediated RNA communication. This system can help us more comprehensively understand the role of animal vectors in virus-host interactions and meanwhile expand our understanding of the new mechanisms of virus-host interactions involving animal viruses or animals. Finally, we summarize the role of artificial intelligence (AI) in predicting virus-host interactions at the molecular level and discuss the therapeutic potential and current challenges of antiviral vaccine development targeting ncRNAs. These insights may provide a novel direction for investigating the interactions between animal viruses and hosts.

## 2 lncRNAs involved in animal virus-host interaction

Long non-coding RNA (lncRNA) is a non-coding transcript with a sequence length greater than 200 nucleotides, lacking or only partially possessing the potential of protein coding (Kapranov et al., 2007). Recent studies have identified lncRNAs as key regulators of host-virus interactions during viral infection, where these RNAs can act as proviral factors or exert antiviral effects. Lnc-ALOX12 has been shown to be specifically upregulated in cells infected with influenza A virus (IAV). Lnc-ALOX12 promotes IAV infection by binding to the IAV RNA polymerase subunit PB2, ensuring PB2 nuclear import and efficient viral RNA synthesis. Importantly, lnc-ALOX12 regulates the function of PB2 in viral RNA synthesis in a species-specific manner, thereby creating a barrier for avian influenza viruses to transmit to humans and playing a role in the cross-species transmission of influenza viruses from avian to mammalian hosts (Wang J. et al., 2025). LncRNAs hold great potential as biomarkers in the process of virus-host interactions. Peng et al. performed whole-transcriptome analysis on four mouse strains to investigate host responses to severe acute respiratory syndrome coronavirus (SARS-CoV) infection, identifying approximately 500 differentially expressed lncRNAs during infection. This study first revealed widespread differential expression of lncRNAs in response to viral infection, suggesting that ncRNAs are involved in regulating host responses and could serve as biomarkers for SARS-CoV infection (Peng et al., 2010). Additionally, Robin et al. compared and analyzed the expression profiles of lncRNAs and mRNAs in hemocytes and fat body of fall armyworm Spodoptera frugiperda infected with the polydnavirus HdIV (Hyposoter didymator IchnoVirus) and densovirus JcDV (Junonia coenia densovirus), revealing that three differentially expressed lncRNAs may serve as potential host genes of microRNAs in response to viral infection, thereby participate in gene regulation (Robin et al., 2023). This study provides potential insights into the role of lncRNAs in antiviral defense in Spodoptera frugiperda, facilitating a better understanding of the biological function of lncRNAs in the interaction between virus and fall armyworm host. The above studies highlight the role of lncRNAs as biomarkers in the context of virus-host interactions. Other multiple studies have also revealed the functions of lncRNAs in animal virus-host interactions, with relevant information summarized in **Table 1**.

**Table 1:** lncRNAs involved in animal virus-host interaction.

LncRNA	Source/host species	Virus/virus family	Direction of effect	Mechanism or pathways	Evidence level	Cell or animal model	Reference
LncRNA ZFAS1	Host-derived /Mouse	Pseudorabies virus (PRV)/neurotropic alpha herpesvirus	Proviral	Knockdown of ZFAS1 modulates microglia-driven inflammation without altering viral replication	A	Mouse	(Phan Van et al., 2025)
LncRNA DARVR	Host-derived /Cell	Rotavirus (RV)	Antiviral	DARVR inhibits RV replication by sponging off miR-365-1-5p and alleviating its suppression of LAMB1 expression/complement C3 pathway	A	MA104 cells	(Song et al., 2025)
LncRNA446	Host-derived /Cell	Porcine epidemic diarrhea virus (PEDV)	Antiviral	lncRNA446/Alix axis can repair intestinal barrier injury after PEDV infection/tight junction (TJ) signaling pathway	A	IPEC-J2 cells	(Qin et al., 2023)
lncRNA TCONS_00054158	Host-derived /Cell	Porcine reproductive and respiratory syndrome virus (PRRSV) /The *Coronavirid-ae* family	Proviral	Regulate apoptosis during PRRSV infection by modulating the expression of TNFSF10/Epstein-Barr virus infection pathway	C	Porcine alveolar macrophages (PAMs)	(Zhang et al., 2017)
LncRNAlinc000889	Host-derived /Cell	Avian reovirus (ARV)/The Arteriviridae family	Antiviral	Inhibits the replication of ARV at the transcriptional, translational levels and viral titer/NLR, RLR, and TLR pathways	B	Duck embryo fibroblasts	(Zhang S. et al., 2025)
LncRNA EDAL	Host-derived /Mouse and cell	Rabies virus (RABV)/Neurotropic virus	Antiviral	Exert antiviral function via blocking O-GlcNAcylation that determines EZH2 lysosomal degradation/lysosomal degradation pathway	A	Mouse and neuronal cells	(Sui et al., 2020)
LncRNA UBC	Host-derived /Cell	Swine hepatitis E virus (SHEV)	Context-dependent	ORF3 protein affects bile secretion by regulating lncRNA UBC network on Bile Secretion Pathway	C	HepG2 cells	(Jiao et al., 2026)
LncRNA-up4	Host-derived /Cell and chicken tissues	H9N2 influenza virus/The Orthomyxoviridae family	Proviral	Silencing lncRNA-up4 impaired IAV replication by upregulating the expression of several critical antiviral molecules, overexpression of lncRNA-up4 increased viral replication/NF-κB signaling pathway	A	DF-1 cells and chicken tissues	(Sajjad et al., 2025)
LncRNA-000819, etc	Host-derived /Cell	Porcine delta coronavirus (PDCoV)/The *Coronavirid-ae* family	Context-dependent	lncRNAs as potential biomarkers of PDCoV infection/metabolic and TNF signaling pathways	C	Swine testicular (ST) cells	(Liu et al., 2019)
LncRNA-SUSAJ1	Host-derived /Cell	Japanese encephalitis virus (JEV)/the family Flaviviridae	Antiviral	Knockdown of CCR1 upregulated lncRNA-SUSAJ1 expression, resulting in resistance to JEV proliferation	C	PK-15 cells	(Zhou et al., 2021)

*A, *in vivo* animal evidence plus mechanistic rescue; B, cell-based gain/loss-of-function plus target validation; C, omics discovery plus qPCR or partial validation; D, prediction or association only.

**Figure 1:**
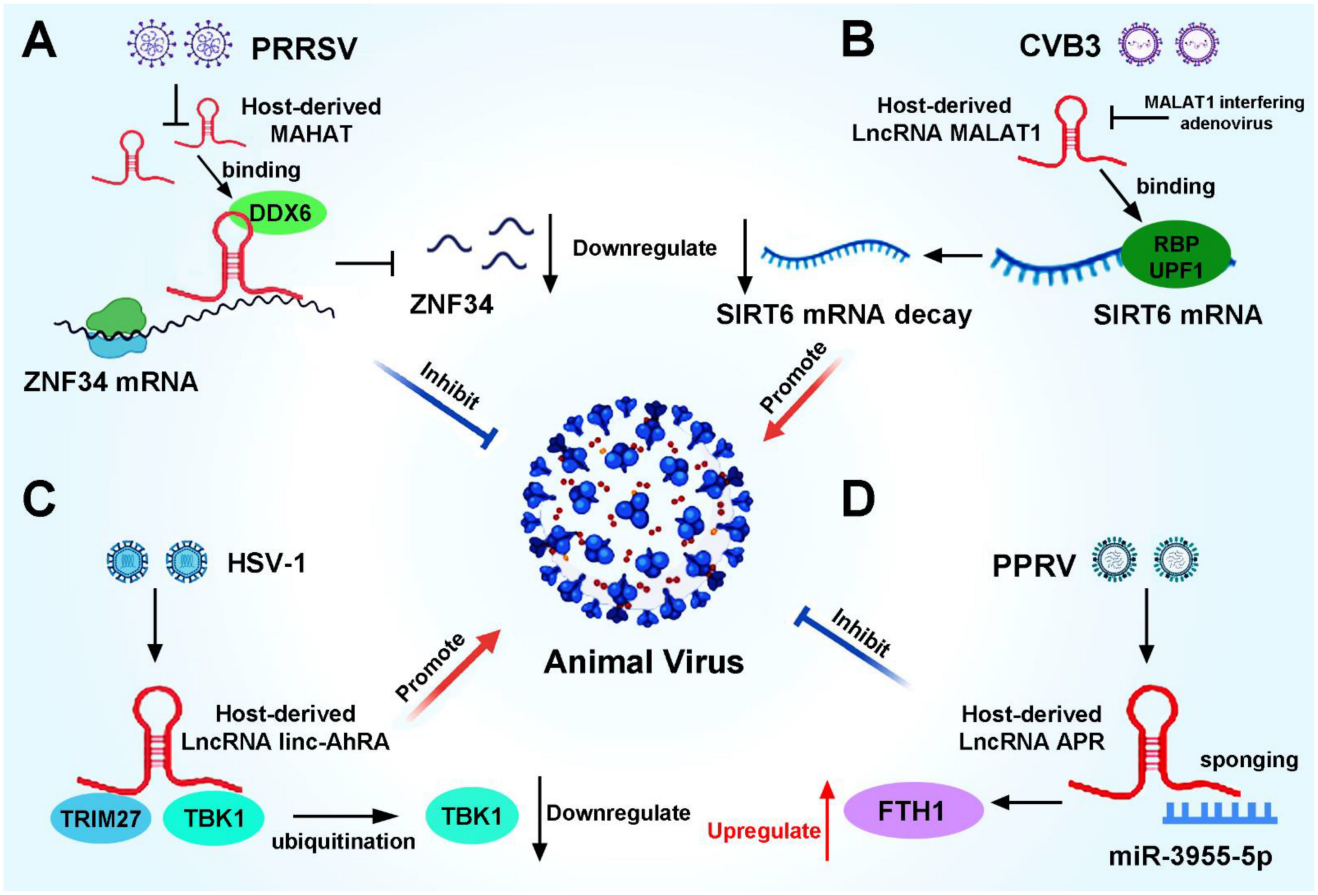
Mechanisms of lncRNA involved in animal virus-host interaction. **(A)** LncRNA MAHAT acts as a regulator and recruits DDX6 to bind to ZNF34 and inhibits replication of PRRSV; **(B)** LncRNA MALAT1 binding RBP UPF1 to mediate SIRT6 mRNA decay and accelerating VMC; **(C)** LncRNA linc-AhRA acts as a scaffold for the binding and interaction of TRIM27 and TBK1; **(D)** LncRNA APR acts as a sponge to prevent miR-3955-5p from inhibiting FTH1.

Many high-impact recent outbreaks and pandemics have involved animal-origin viruses (Crits-Christoph et al., 2024), due to the high variability of viruses and the lack of specific therapeutic options, large-scale global pandemics frequently emerge. In the field of molecular biology, lncRNAs have emerged as key players in the pathogenic mechanisms of zoonotic viruses affecting. In this review, we uncovered four common functions of lncRNAs and elucidate their mechanisms in mediating pathogen-host interactions (**Figure 1**). Firstly, porcine reproductive and respiratory syndrome virus (PRRSV) has caused enormous economic losses to the global swine industry. Liu et al. discovered a novel lncRNA, named MAHAT, which negatively regulates the expression of *ZNF34* by recruiting and binding to DDX6, an RNA helicase that forms a complex with *ZNF34*, ultimately leading to the reduction of PRRSV replication (Liu Y. et al., 2022) (**Figure 1A**). This finding reveals a novel mechanism by which PRRSV evades the host antiviral innate immune response by downregulating the MAHAT-DDX6-ZNF34 pathway. MAHAT could be a host factor target for antiviral therapies against PRRSV infection. Secondly, coxsackievirus B3 (CVB3) is the primary pathogenic agent of viral myocarditis (VMC). Recent studies have revealed that lncRNA MALAT1 accelerates the progression of VMC by enhancing the recruitment of UPF1 to *SIRT6* mRNA and destabilizing its stability in a mouse model of VMC infected with CVB3 (Zeng et al., 2024) (**Figure 1B**). This discovery identified MALAT1 as a new positive regulator of viral myocarditis, which can promote inflammation and myocardial dystrophy in myocardial tissues and cells in mice with VMC. It also provides us with the possibility of enhancing protective antiviral immunity by inhibiting MALAT1 expression. Thirdly, lncRNAs function as scaffolds to assist molecules in performing their functions. Wang et al. found that lncRNA linc-AhRA (activated by aryl hydrocarbon receptor (AhR)) acts as a molecular scaffold binds to both tripartite motif-containing 27 (TRIM27) and TANK-binding kinase 1 (TBK1), which enhances TRIM27-TBK1 interaction in infected with Herpes simplex virus type-1 (HSV-1) (Wang et al., 2021). This interaction promotes TRIM27-mediated ubiquitination of TBK1 and leads to TBK1 degradation, ultimately inhibiting the production of type I interferon (I-IFN) and limiting the innate immune response of microglia to neurotropic herpesvirus infection (**Figure 1C**). This study demonstrates that linc-AhRA is a negative regulator of I-IFN production in microglia and provides a strong foundation for the development of nucleotide drugs based on lncRNAs. Fourthly, peste des petits ruminants virus (PPRV) is a significant pathogen that has long posed a major threat to the productivity of small ruminants worldwide. It has been reported that host factor lncRNA APR promotes ferritin-heavy chain 1 (FTH1) expression by sponging miR-3955-5p, thereby attenuating PPRV infection-induced ferroptosis and suppressing PPRV infection (Wen et al., 2025) (**Figure 1D**). This study provides us with new insights into PPRV-host cell interaction and pathogenesis and provides a potential therapeutic target for antiviral intervention. Moreover, Chang et al. found a new lncRNA (named MIR2187HG), which can produce miR-2187-3p by acting as a precursor of miR-2187-3p and inhibit the intracellular anti-virus signaling pathway mediated by TANK-binding kinase 1(TBK1), finally promoting Siniperca chuatsi rhabdovirus (SCRV) infection (Chang et al., 2022). This discovery is the first time to identify the molecular regulatory function of lncRNA as a pre-miRNA in teleost fish, providing new insights into the lncRNA-mediated negative feedback regulation of the antiviral innate immune response in fish, and enriching our understanding of host-virus interactions.

In summary, lncRNAs play a critical role in animal virus-host interactions and can act as potential therapeutic targets for the treatment of viral infections. They can also serve as relevant prognostic indicators for clinical viral infection. In the future, research on lncRNAs in animal viruses and their hosts will become an emerging research direction in the field of virology.

## 3 miRNAs involved in animal virus-host interaction

**Figure 2:**
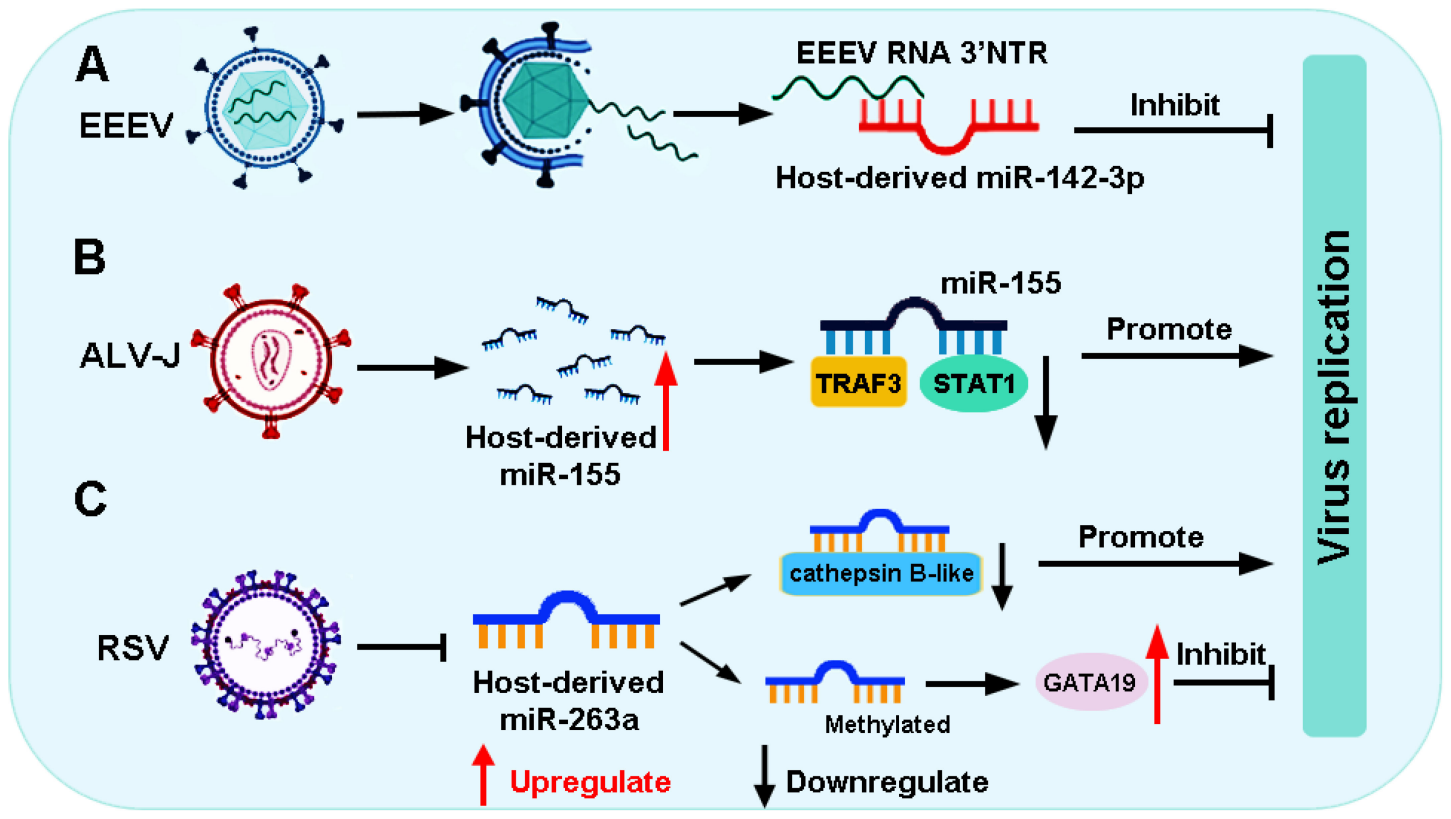
Mechanisms of miRNA involved in animal virus-host interaction; **(A)** miR-142-3p effectively restricts the replication of EEEV by binding to sites in the 3’non-translated region (NTR); **(B)** miR-155 promotes ALV-J replication by dually targeting TRAF3 and STAT1; **(C)** MiR-263a promotes RSV replication by targeting cathepsin B-like gene or inhibits RSV by being methylated to promote GATA19 expression.

MicroRNA (miRNA) is a class of small non-coding RNAs approximately 19–24 nucleotides (nt) in length and belong to RNA interference (RNAi) pathway, which is one of the most important antiviral immune systems in animals, especially insects (Nayak et al., 2013). Cellular miRNA plays a key role in virus-host interactions, functioning as a post-transcriptional regulator to guide the regulation process of virus proliferation (Wong et al., 2020). Any understanding of the potential role of miRNAs in virus-host interactions requires an appreciation of how miRNAs are transcribed and processed and how they normally function in animal (Bartel, 2004). In this review, we will focus on the role of animal viral miRNAs in virus-host interactions and discuss the role of host miRNA expression during viral infection of hosts. Moreover, we also review the miRNA in the plant virus-insect vector-plant host system as a special cross-kingdom RNA trafficking pattern (**Figure 2**).

The expression of host miRNAs can be altered by viral infection. Additionally, miRNAs can mediate viral replication in the host by complementing the viral genome or by indirectly binding to key host genes. For instance, Trobaugh et al. found that miR-142-3p effectively restricts the replication of mosquito-borne North American (NA) Eastern Equine Encephalitis Virus (EEEV) in myeloid cells by binding to sites in the 3’non-translated region (NTR) of the RNA genome (Trobaugh et al., 2014) (**Figure 2A**). Furthermore, their research highlights that the miR-142-3p targeting region is crucial for effective viral infection in mosquito vectors. Understanding miRNA expression levels and viral genotypes could provide new insights into individual host variation in EEE and other potential RNA virus diseases. Moreover, another virus, avian leukosis virus J subgroup (ALV-J), causes significant economic losses in poultry by inducing immunosuppression and myelocytomas (Payne and Nair, 2012). Zhang et al. latest study reveals that ALV-J infection significantly activates miR-155 expression, and miR-155 suppresses IFN-β production and signaling by dually targeting TRAF3 and STAT1, thereby promoting ALV-J replication in chicken embryo fibroblasts (CEF). The discovery of this positive feedback loop uncovers a novel mechanism by which ALV-J exploits miRNA to evade host immune responses, providing deeper insights into the pathogenesis of ALV-J (Zhang et al., 2025b) (**Figure 2B**).

Moreover, there is a special class of miRNAs that can spread not only within the same species but also across species. Instances of cross-species miRNA transfer regulating viral infections in hosts or insect vectors have been documented in the literature (Hussain et al., 2012), yet, how they act as cross-kingdom effectors influencing the tripartite relationship among viruses, vectors, and hosts remains largely elusive. Here, we describe it as cross-kingdom RNA trafficking in a plant virus-insect vector-plant host system. For example, the latest research by Zhao’s team revealed the dual role of the evolutionarily conserved insect microRNA, miR-263a, in regulating rice stripe virus (RSV) infection in both the insect vector, small brown planthopper and rice host. In the midgut epithelial cells of small brown planthopper (SBPH), miR-263a inhibits cell apoptosis by targeting the cathepsin B-like gene, thereby promoting the accumulation of RSV. Upon transmitted to rice, miR-263a is methylated, leading to up-regulated expression of GATA19, thus activating jasmonate signaling pathway and enables rice to develop resistance to RSV (Zhao et al., 2025b) (**Figure 2C**). This study shows that cross-species transmission of miRNA cannot only regulate the apoptosis activity in insect cells, but also be transported into rice cells, thereby profoundly influencing the interactions among the virus, vector and host. Finally, we have detailed the latest research findings on the functional roles of miRNAs in animal virus-host interactions in **Table 2**.

**Table 2:** miRNAs involved in animal virus-host interaction.

miRNA	Source/host species	Virus/virus family	Direction of effect	Mechanism or pathways	Evidence level	Cell or animal model	Reference
miR-329	Host-derived /Mouse and cells	Herpes simplex virus type-1 (HSV-1)	Proviral	miR-329 functions as a pro-viral miRNA by disrupting TLR9 signaling, thus facilitating HSV-1 replication/TLR and interferon signaling pathways	A	C57BL/6 mice MEFs and HCEs	(Sharma et al., 2025)
miR-KT-635	Virus-derived /Cell	Cyprinid herpesvirus 2 (CyHV-2) /Family Alloherpesviridae	Proviral	Overexpression of CyHV-2-KT-635 mimics led to a decrease of orf121 protein levels, while CyHV-2-KT-635 inhibitor resulted in an increase of orf121 protein levels/JAK-STAT pathway, MAPK pathway	C	GiCF cells	(Luo et al., 2021)
Osa-miR159a.1-1 Osa-miR167a	Host-derived /Rice	Rice stripe virus (RSV)	Osa-miR159a.1-1 (Proviral) Osa-miR167a (Antiviral)	Osa-miR159a.1–1 promotes RSV replication by enhancing mRNA stability and Osa-miR167a directly binds to the RSV RdRp to inhibit viral replication/Notch signaling pathway	A	Small brown plantho-pper /Rice	(Wang Q. et al., 2025)
miR-H1/H6	Virus-derived /Cell and mouse, rabbit	HSV-1	Proviral	The absence of miR-H1/H6 seed sequences leads to a significant reduction in HSV-1 reactivation in mouse and rabbit models/canonical miRNA biogenesis pathway	A	Mouse and rabbit /Cell	(Barrozo et al., 2020)
bta-miR-11985/1281/12034/let-7i/17-5p	Host-derived /Cattle	Lumpy skin disease virus (LSDV)	Antiviral	These findings provide significant insights into the immune and protective responses during LSDV infection in cattle/MAPK/Rap1/PI3K-Akt/mTOR signaling pathways	C	Cattle	(Truong et al., 2025)
ISKNV-miR-c17-5p/-c19	Virus-derived /Cell	ISKNV /The Megalocytivirus genus	Antiviral	Inhibitions of ISKNV-miR-c17-5p and -c19 on MCP expression were consistent with those on the virus titer in cell supernatants	C	MFF-1 cells	(He et al., 2020)
dev-miR-D28-3p	Virus-derived /*α-herpesvirus* family	Duck plague virus (DPV)	Antiviral	Dev-miR-D28-3p inhibits viral replication via targeting UL27	B	duck embryo fibrobla-sts (DEFs)	(Ni et al., 2024)
miR-M4-5p	Virus-derived /Cell	Marek’s disease virus (MDV) /Herpesvirus	Proviral	miR-M4-5p mimics reduced the luciferase level by 40% in CEF, indicated that exosome-delivered viral miRNA is functional in the recipient CEF cells/TGF-beta signaling pathway	C	MSB-1 cells and CEF	(Teng et al., 2024)
miR-211-5p/320-5p/7049-5p	Host-derived /Cell	Mouse mammary tumor virus (MMTV)miR-211-5p/7049-5p (Antiviral) miR-320-5p (Proviral)	miR-211-5p/7049-5p (Antiviral) miR-320-5p (Proviral)	miR-320-5p exhibited an increase in expression, miR-7049-5p and miR-211-5p showed a decrease upon MMTV expression/PI3K/Akt signaling pathway	C	HC11 cells	(Gull et al., 2024)
miR-203/136/188-3p/21-3p/141	Host-derived /Animal and cells	Highly pathogenic avian influenza (HPAI) H5 viral /Orthomyxoviridae family	miR-203/136/188-3p (Antiviral) miR-21-3p/141 (Proviral)	These miRNAs can influence IFN signaling, DNA demethylation, cytokine production, and viral RNA replication/IAV replication pathway, TGF-β, MAPK, and TLR signaling pathways	D	A549, NCI-H292, Vero cells and chicken	(Chowdhury et al., 2024)

*A, *in vivo* animal evidence plus mechanistic rescue; B, cell-based gain/loss-of-function plus target validation; C, omics discovery plus qPCR or partial validation; D, prediction or association only.

However, miRNA regulation is mutual, viruses utilize their own encoded miRNAs to regulate viral gene expression and influence host gene expression, while host miRNAs in turn affect the viral life cycle. Future extensive research on miRNAs related to animal viruses, such as the biogenesis and functional mechanisms of miRNAs in virus-host interactions and the identification of specific miRNAs during viral cross-species transmission and then discover new diagnostic and prognostic markers and therapeutic targets, will be a hot topic in the field of animal virology.

## 4 circRNAs involved in animal virus-host interaction

Circular RNA (circRNA) is an emerging family of RNA originating from non-continuous back-splicing of exons and/or introns in precursor messenger RNA (pre-mRNA), lacking a 3’polyadenylation (poly(A)) tail or a 5’cap structure. CircRNA was initially considered a non-coding RNA that regulates the activity of miRNAs and RNA-binding proteins (Liu and Chen, 2022; Liu X. et al., 2022), which has been shown to affect various aspects of viral infection, including viral replication and pathogenesis (Cadena and Hur, 2017). Since the first animal virus with a circular RNA genome, Hepatitis Delta Virus (HDV), was identified, increasing research has emerged on the functional roles of circular RNAs in animal virus-host interactions (Kos et al., 1986; Limkul et al., 2025). Elucidating the relationship between circRNAs and viruses will provide new insights into viral diseases and may reveal circRNAs as potential antiviral targets.

**Figure 3:**
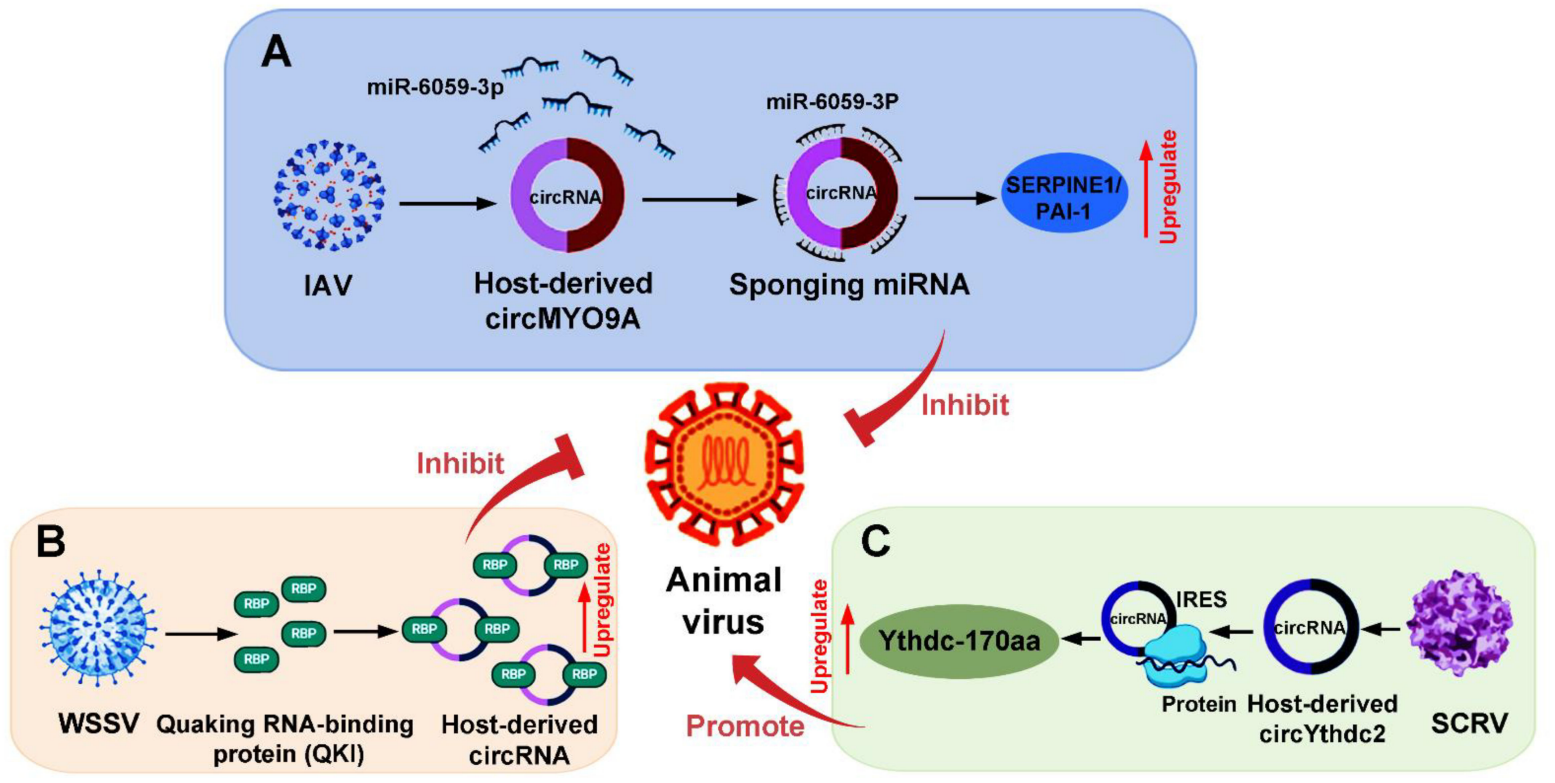
Mechanisms of circRNA involved in animal virus-host interaction; **(A)** circMYO9A up-regulates the expression of SERPINE1/PAI-1 by sponging miR-6059-3p and inhibits the replication of influenza A virus (IAV); **(B)** WSSV promotes QKI expression, then upregulates circRNA and improves antiviral ability; **(C)** circYthdc2 improves the replication ability of SCRV virus by promoting the expression of Ythdc2-170aa mediated by IRES.

Recently, an increasing number of studies have demonstrated that various circRNAs are involved in regulating viral infection and antiviral innate immunity (Teng et al., 2023; Limkul et al., 2025). Differentially expressed circRNAs modulate antiviral innate immunity through various mechanisms. These functions include acting as miRNA sponges to regulate host gene expression, interacting with RNA-binding proteins (RBPs) to fine-tune their activity, and encoding functional peptides with antiviral or pro-viral properties. Here, we will summarize how circRNAs derived from the host and the virus can either restrict or promote viral infection (**Figure 3**). miRNA sponging is one of the functions of circRNAs, which can inhibit miRNA function by directly or indirectly binding to target miRNAs. Zheng et al. recently discovered a novel differentially expressed circular RNA-circMYO9A. Mechanistically, circMYO9A upregulates SERPINE1/PAI-1 expression by sponging miR-6059-3p, thereby inhibiting the replication of influenza A virus (IAV) and subsequently reducing the infectivity of progeny viruses (Zheng et al., 2025) (**Figure 3A**). This finding demonstrates that circMYO9A significantly suppresses viral replication in the lungs of infected mice, confirms the critical role of circRNA in inhibiting IAV replication, and provides novel insights into potential therapeutic strategies involving circRNAs. Moreover, the interaction between circRNA and RBP plays a crucial role in circRNA biogenesis, transcriptional regulation, and viral replication. For example, Phiwthong et al. recently identified a Quaking RNA-binding protein (QKI) in Litopenaeus vannamei, named LvQKI. They further found that silencing LvQKI significantly downregulated circRNA expression, leading to higher viral loads and mortality in shrimp infected with white spot syndrome virus (WSSV). Conversely, introducing recombinant LvQKI protein increased circRNA expression and enhanced antiviral defense, significantly improving survival rates (Phiwthong et al., 2025) (**Figure 3B**). This study demonstrates that LvQKI is a key factor in shrimp circRNA biogenesis and immune defense, highlighting the interaction between LvQKI and circRNA in host defense against viral invasion, and suggesting its potential utility in developing strategies to enhance disease resilience in aquaculture. Furthermore, certain circRNAs can undergo internal ribosome entry site (IRES)-or *N*^6^-methyladenosine (m^6^A)-mediated translation to produce functional peptides (Sun et al., 2021; Zhang et al., 2022). For instance, Zheng et al. found a novel conserved circYthdc2 in vertebrates produced a 170 amino acid polypeptide (Ythdc2-170aa) through IRES/m^6^A dependent translation. Ythdc2-170aa can suppress the host’s antiviral immune response by inhibiting STING, thereby promoting RNA virus replication (Zheng et al., 2024) (**Figure 3C**). This may represent a self-protection mechanism in virus-host interactions. Interestingly, when circYthdc2 is highly expressed, Ythdc2 preferentially promotes the degradation of circYthdc2, thereby further inhibiting the degradation of STING and stabilizing the host’s antiviral immune response, which is of significant importance for further elucidating the functional roles of circRNAs in vertebrate viruses. Similarly, another circRNA-390, encoded by Bombyx mori nucleopolyhedrosis virus (BmNPV), contains an open reading frame (ORF) and four viral IRES elements, and can be translated into an 81-amino-acid viral peptide (VSP81), thereby enhancing viral replication (Zhang et al., 2025a). These findings have the potential not only to deepen our understanding of viral pathogenesis, but also to identify promising targets for the development of novel antiviral therapies.

Of course, an increasing number of circRNAs are being discovered and characterized for their important roles in animal virus–host interactions. We have collected more research on circRNA in animal virus and host in **Table 3**. In summary, elucidating the mechanisms of circRNA in viral infection of host cells and host antiviral immune responses, while identifying novel therapeutic targets, is crucial for effectively mitigating the social and economic burdens caused by viral infections.

**Table 3:** circRNAs involved in animal virus-host interaction.

circRNA	Source/host species	Virus/virus family	Direction of effect	Mechanism or pathways	Evidence level	Cell or animal model	Reference
circEgg	Host-derived /Cell	Bombyx mori cytoplasmic polyhedrosis virus (BmCPV)	Antiviral	circEgg inhibits BmCPV infection by enhancing H3K9ac levels through the circEgg-bmo-miR-3391-5p-Rpd3 network or reducing H3K9me3 levels via circEgg-P122	B	BmN cells	(Qiu et al., 2025)
circ-udg	Virus-derived /Cell	Cyprinid herpesvirus 2 (CyHV-2) /The family *Alloherpesviridae*	Proviral	circ-udg could encode a truncated UDG protein, circ-udg-P147, which consists of 147-amino-acid residues. circ-udg-P147 could increase the level of UDG protein to promote CyHV-2 replication	B	EPC cells	(Zhu et al., 2022)
circ-Vav3	Host-derived /Cell	Avian leukosis virus subgroup J (ALV-J)/Retrovirus	Proviral	circ-Vav3 inhibits ALV-J-induced autophagy through the gga-miR-375/CIP2A/AKT pathway	A	DF-1 cells	(Chen et al., 2025)
circMORC3	Virus-derived/Cell	Siniperca chuatsi rhabdovirus (SCRV)	Proviral	circMORC3 and MORC3 can synergistically inhibit TRIF and IRF3, promoting viral immune escape/IRF3 and NF-κB signaling pathways	B	MsbC and EPC cells	(Wang et al., 2023)
circRNA_1193	Host-derived /Cell	BmCPV	Antiviral	Overexpression of circRNA_1193 suppressed BmCPV replication, whereas its knockdown increased viral replication/immune pathways	A	BmN cells	(Li et al., 2025)
circLSM14A	Host-derived /Cell	PEDV /The Coronavirida-e family	Proviral	circLSM14A inhibits miR-27b-5p activity by acting as a sponge, thereby increasing HMGB1 expression and ultimately enhancing PEDV replication/HMGB1/TLR4/NF-κB signaling pathway	B	Vero cells	(Wang Y. et al., 2025)
circRNA_8521	Host-derived /Cell	Senecavirus A (SVA) /The Parvoviridae family	Proviral	circ_8521 functioned as miR-324 sponge and promoted LC3A expression and ultimately SVA infection	B	PK-15 cell	(Yang et al., 2024)
circ_6789/6793/6785/6798/6791/6794/3119	Host-derived /Dog	Canine parvovirus (CPV)/The Parvoviridae family	Proviral	These circRNAs hold potential as clinical biomarkers for assessing CPV prognosis, organ damage and therapeutic responses/inflammatory pathways	C	Dogs	(Degirmencay et al., 2025)
circPDCD4	Host-derived /Cell	Porcine circovirus type 2 (PCV2)/Circoviridae family	Antiviral	PCV2 induced miR-21 by directly inhibiting PDCD4 and circPDCD4, aggravating the production of PCV2-induced inflammatory mediators/NF-κB pathway	B	PK-15 cells	(Li et al., 2021)
circPTPN3	Host-derived /Duck	Duck hepatitis A virus type 3 (DHAV-3)/Highly pathogenic picornavirus	Proviral	circPTPN3 promotes DHAV-3 replication by sponging antiviral miR-130b-5p to enhance proviral gene expression/metabolic and immune-related pathways	A	Duck embryo hepatic-ytes (DEHs)	(Liu et al., 2025)

*A, *in vivo* animal evidence plus mechanistic rescue; B, cell-based gain/loss-of-function plus target validation; C, omics discovery plus qPCR or partial validation; D, prediction or association only.

## 5 Other ncRNAs with emerging-evidence in animal virus–host interactions

Compared to lncRNA, miRNA and circRNA, there have been fewer research reports on tsRNA, snoRNA, piRNA, sfRNAs and vsiRNAs in animal virus-host interactions in recent years. In this review, we provide a classified summary of these ncRNAs and focus on sfRNAs and vsiRNAs.

Transfer RNA (tRNA)-derived small RNAs (tsRNAs), also known as tRNA-derived fragments (tRFs), represent a novel class of ncRNAs that are generated through the specific cleavage of precursor or mature tRNAs (Kim et al., 2017). In particular, tsRNAs have been associated with viral infections (Wang et al., 2013). Taxis et al. detected the expression changes of ncRNAs by extracting the serum RNA of bovine viral diarrhea virus (BVDV) infected cattle, and found that 5’tsRNA-Gly-CCC and tsRNA-Gly-GCC in infected group were significantly lower than those in control group (Taxis et al., 2019). This finding preliminarily indicates the importance of tsRNA in viral infections. However, further research is needed to support the expression changes of 5’tsRNA-Gly-CCC and tsRNA-Gly-GCC in BVDV-infected animals. Small nucleolar RNAs (snoRNAs) are small ncRNAs ranging from 60 to 300 nucleotides in length and are conserved across various eukaryotes. They constitute a relatively large component of chromatin-associated RNAs (caRNAs) in insect and mammalian cells (Lafontaine and Tollervey, 1998; Dupuis-Sandoval et al., 2015). Moreover, snoRNAs are often dysregulated during viral infections, and many have been found to participate in viral replication or virus-host interactions (Stamm and Lodmell, 2019). A study demonstrated that *snoRNA:U3:9B* is significantly upregulated following Sindbis virus infection, and deletion of the its gene in combination with Sindbis virus infection is lethal. Further investigation revealed that *snoRNA:U3:9B* is essential for recruiting the chromatin remodeler Brahma to target immune genes (Jain et al., 2025). This result reveals an antiviral defense mechanism that depends on snoRNA recruiting chromatin remodeling factors to immune genes to promote antiviral gene activation. PIWI-interacting RNAs (piRNAs) are the most abundant class of non-coding RNA, slightly longer than siRNA and miRNA, with an average length ranging from 23 to 31 nucleotides (Siomi et al., 2011). In animals, piRNA, which are 21–35 nucleotides in length, can silence transposable elements, regulate gene expression, and defend against viral infections (Ozata et al., 2019). For instance, Sun et al. found that domestic chickens can defend against the avian leukosis virus (ALV) by activating piRNAs to target transposable elements (TEs) (Sun et al., 2017). Their findings identify new piRNA acquisition patterns by activating pre-existing genomic loci. Furthermore, piRNA pathway protein knockdown leads to enhanced production of Semliki Forest virus (SFV) in mosquito cells. piRNA pathway is considered an additional small RNA-mediated antiviral response to the known infection-induced siRNA response, and arbovirus infection can trigger the piRNA pathway in mosquito cells, and knocking out piRNA proteins enhances viral production (Schnettler et al., 2013).

Subgenomic flaviviral RNA (sfRNA) is an RNA decay product, resulting from partial degradation of the viral RNA genome (gRNA) by 5’-3’exoribonucleases which stall on secondary structures in the 3’UTR (Pijlman et al., 2008). sfRNAs are produced during flavivirus infections in both arthropod and vertebrate cells and critical for the efficient viral replication (Liu et al., 2023). Flaviviruses are emerging and reemerging RNA viruses that include important human pathogens, such as Zika (ZIKV), dengue (DENV), yellow fever and JEV (Teramoto et al., 2023). ZIKV is an emerging mosquito-borne flavivirus that causes severe outbreaks in human populations. Pallarés et al.’s latest study confirms that ZIKV has evolved a mechanism to reprogram antiviral pathways into a proviral process. They found that ZIKV sfRNA upregulates PKR expression by promoting PKR autophosphorylation, significantly reduces the translational efficiency of type I interferons and interferon-stimulated genes, and ultimately augments virion production (Pallares et al., 2024). This result reveals a novel virus adaptation mechanism mediated by sfRNAs, where ZIKV sfRNAs increases its fitness by repurposing the antiviral role of PKR into a proviral factor. Viruses hijack extracellular vesicles (EVs) to facilitate their dissemination. However, the mechanisms underlying the selective packaging of viral RNA into EVs remain unclear. Rachenne and his team utilized DENV virus to secrete sfRNA, which enhances transmission, into mosquito saliva EVs to investigate the mechanism of viral RNA loading. They found that DENV sfRNA harnesses mosquito Syntenin homolog (AeSyntenin) to induce the secretion of sfRNA-loaded cells and salivary exosomes (Rachenne et al., 2026). Their results reveal a molecular mechanism for viral RNA packaging into EVs.

During virus infection, viral double‐stranded RNAs are cleaved to form virus‐derived small interfering RNAs (vsiRNAs) 21 to 24 nucleotides (nt) in length by Dicer‐like enzymes (Weiberg et al., 2013). VsiRNAs play important roles in regulating host gene expression to promote virus infection and induce RNA silencing to suppress virus infection. For example, Wu et al. research discovered that infection of macrophages with wild-type coronavirus (infectious bronchitis virus, IBV) and influenza virus (avian influenza virus, AIV) can trigger RNAi antiviral immunity and produce a certain amount of virus-derived siRNA (vsiRNA). Further studies also revealed that the combination of PS-vsiRNA plasmid and nanomaterial polyetherimide (PEI) exhibits excellent antiviral activity in specific pathogen-free (SPF) chickens (Wu et al., 2025). These findings indicate that RNA viruses trigger the production of vsiRNA in avian somatic cells, providing an important theoretical basis for the management and prevention of different RNA viruses in birds, and are of great significance for the application of therapeutic vaccines. Moreover, vsiRNAs can also play a positive role in viral infection. A recent study by Zhao et al. found that three vsiRNAs derived from the terminal panhandle structure of rice virus promote viral infection in insect vectors and host plants. These vsiRNAs synergistically downregulate the expression of *DOPA* decarboxylase to inhibit the melanization immune response in insect vectors. They also found that viral titer decreased significantly in vsiRNA-silenced transgenic rice, indicating that vsiRNA promotes the replication of RSV in rice (Zhao et al., 2025a). This study clarifies that vsiRNAs derived from the conserved panhandle structure of negative-sense RNA viruses play a conserved positive role in viral infection. Overall, these ncRNAs, especially sfRNA and vsiRNAs, play critical roles during virus-host interactions, and the functional mechanisms of sfRNA and vsiRNAs in animal/plant hosts and insect vectors will be further revealed in the future.

## 6 Crosstalk between ncRNAs in animal virus-host interaction

**Figure 4:**
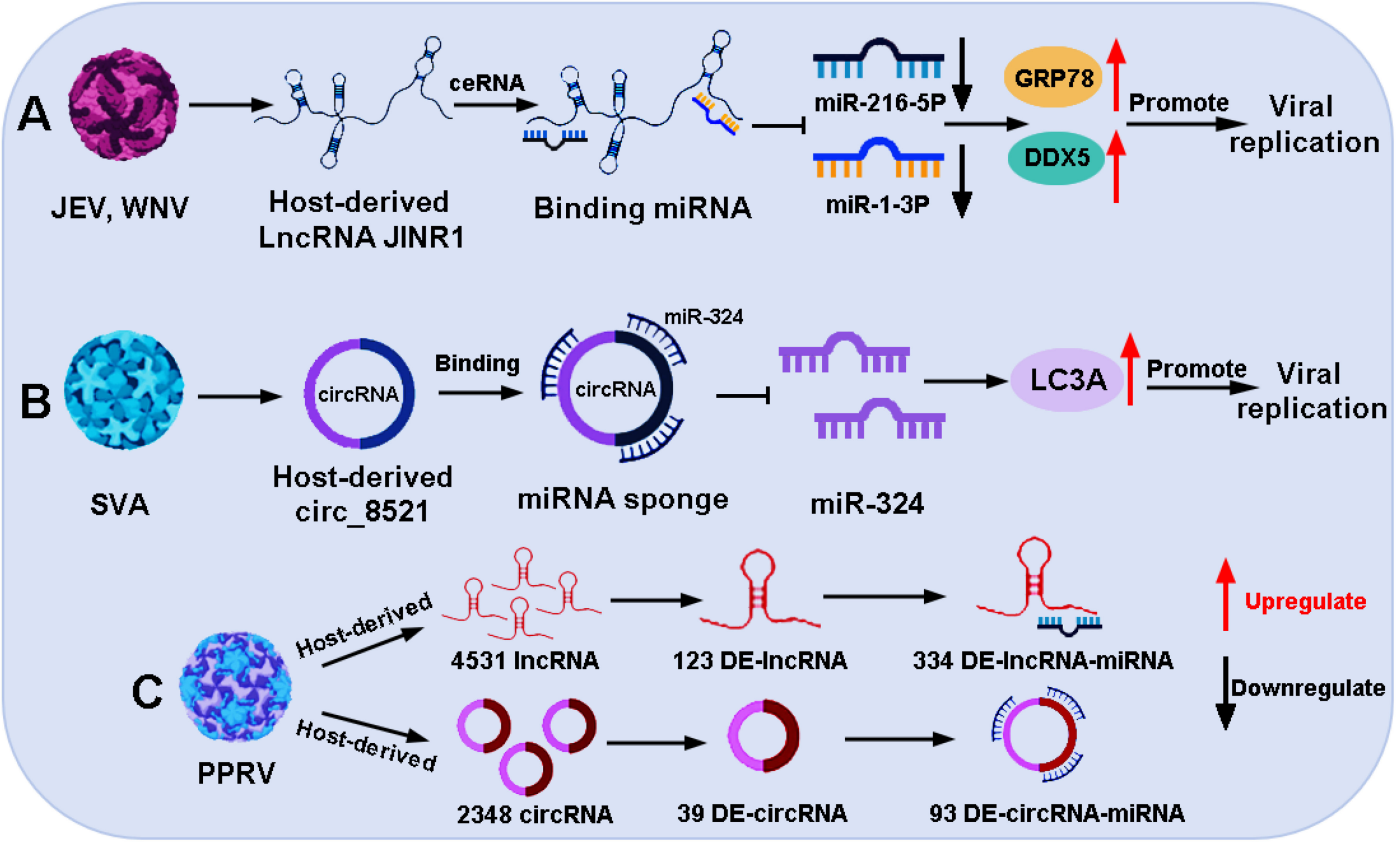
Crosstalk between ncRNAs in animal virus-host interaction. **(A)** LncRNA JINR1 promotes viral infection and regulates the expression of GRP78 and DDX5 by directly binding with miR-216b-5p/1-3p; **(B)** Circ_8521 promotes SVA infection by up-regulating LC3A in combination with miR-324; **(C)** CeRNA network analysis of lncRNAs and circRNAs of common miRNA targets in B95a cells infected by PPRV.

In recent years, interaction networks composed of ncRNAs (including lncRNAs, miRNAs, and circRNAs) have attracted increasing attention due to their important roles in various biological processes such as viral infection (Li et al., 2021; Peng et al., 2023). A deeper understanding of the crosstalk mechanisms of ncRNAs in virus-host interactions, and elucidating the impact of ncRNAs on host-virus interactions will help us better understand viral pathogenesis and develop novel therapeutic interventions (**Figure 4**).

LncRNAs can function as a competing endogenous RNA (ceRNA) by binding to and sequestering miRNA, thereby reducing miRNA-mediated post-transcriptional gene silencing. LncRNAs regulate viral replication and host interactions by modulating gene expression at epigenetic, transcriptional, and post-transcriptional levels (Salmena et al., 2011; Meng et al., 2017). JEV and West Nile virus (WNV) are dual-host viruses that are horizontally transmitted between vertebrate hosts and arthropod vectors (e.g., mosquitoes and ticks), and their infection can lead to neuroinflammation and neuronal cell death (Pierson and Diamond, 2020). Tripathi et al. report that JEV/WNV infection promotes the expression of JEV-induced LncRNA JINR1 and suppresses the expression of miR-216b-5p/1-3p; meanwhile, it was found that depletion of JINR1 during viral infection prevents the downregulation of miR-216b-5p/1-3p (**Figure 4A**). Mechanistically, LncRNA JINR1 acts as a competing endogenous RNA (ceRNA) that directly binding to miR-216b-5p/1-3p, leading to the upregulation of their target genes glucose-regulated protein 78 (GRP78) and DEAD-box helicase 5 (DDX5), respectively, thereby promoting viral infection (Tripathi et al., 2025). This study indicates that lncRNA JINR1 is a key proviral factor for JEV/WNV infection and could serve as a potential therapeutic target for developing anti-viral against virus infection. In addition to lncRNA-miRNA interaction, circRNA, also as ceRNA, competitively binding to miRNA and regulating the expression level of miRNA target mRNA. For instance, SVA can cause vesicular disease in pigs, imposing a significant economic burden on the swine industry. Yang et al. found that circ_8521 is significantly upregulated in SVA-infected cells and promotes SVA infection (Yang et al., 2024). Further study revealed that circ_8521 enhances the expression of microtubule associated protein 1 light chain 3 alpha (LC3A) by binding to miR-324 (**Figure 4B**). Their finding demonstrated that circ_8521 acts as an endogenous miR-324 sponge to sequester miR-324, thereby promoting LC3A expression and ultimately facilitating SVA infection. It also suggests that circ_8521 might be a potential therapeutic target to inhibit SVA infection. Moreover, lncRNA and circRNA are becoming increasingly important due to their critical roles in various biological processes, including their newly discovered functions in regulating virus-host interactions. For example, lncRNA MALAT1 encodes a 52-amino acid micropeptide (miPEP-52) that promotes influenza A virus replication by suppressing innate immune responses (Rai et al., 2026). These findings, combined with our review of MALAT1 in the section “lncRNAs Involved in Animal Virus-Host Interaction”, collectively demonstrate that MALAT1 MALAT1 may act through RNA-level mechanisms in some infections and through encoded micropeptides in others.

Genome-wide expression studies have become important tools to understand host-virus interactions. Tanuj et al. performed a genome-wide analysis of lncRNAs and circRNAs in B lymphoblasts (B95a) from healthy and PPRV-infected marmosets, identified differentially expressed ncRNAs, and proposed a ceRNA network that may function in PPRV-host interactions. They found that a total of 4531 lncRNAs and 2348 circRNAs were expressed in both mock and PPRV-infected samples, with 123 lncRNAs and 39 circRNAs significantly dysregulated (Tanuj et al., 2022). Further functional mechanism analysis via a ceRNA network identified lncRNAs, circRNAs, and mRNAs that share common miRNA targets (**Figure 4C**). Specifically, their findings may provide insight for better understanding of animal virus PPRV-host interactions. Moreover, in the early stage of JEV infection in mouse brain tissue and mouse microglia, Chen et al. identified 61 differentially expressed circRNAs, 172 differentially expressed miRNAs and 706 differentially expressed mRNAs via high-throughput sequencing and statistical analysis. Further studies revealed the circRNA-miRNA regulatory network targeting CX3CR1, and confirmed the inhibitory effect of this regulatory network on JEV (Chen et al., 2023). Their study proposed a novel ceRNA pathway that affects JEV infection both *in vivo* and *in vitro*, which may serve as a therapeutic target against JEV.

In summary, lncRNA/circRNA-miRNA-mRNA interactions may represent a crucial pathway in the pathogenesis of animal viruses. Elucidating the impact of host and viral non-coding RNAs on host-virus interactions will enhance our understanding of viral pathogenic mechanisms and provide a theoretical foundation for developing novel viral vaccines and therapeutic targets.

## 7 Conclusions and future perspectives

In recent years, the importance of ncRNA-mediated virus-host interactions has been increasingly revealed. Studies show that both viral ncRNAs and host ncRNAs can play important roles in promoting viral replication and inhibiting viral infection by regulating the expression of key target genes. In this review, we summarize the functions of lncRNAs, miRNAs, circRNAs and other ncRNAs (sfRNAs and vsiRNAs) in virus-host interactions and highlight their molecular mechanisms. Primary mechanisms-including lncRNAs function as scaffolds, circRNAs act as sponge and their crosstalk with miRNAs. Secondly, ncRNAs play a crucial role in regulating translation initiation and mRNA stability of key genes during virus-host interactions. Lastly, ncRNA networks (including lncRNAs, circRNAs, and the miRNA-mRNA axis) orchestrate the entire cascade of virus-host interactions, wherein both viral and host ncRNA networks modulate infection efficiency or suppress viral replication capacity by regulating target genes. Moreover, ncRNA-based immunotherapies are increasingly being applied to the protection of humans, animals and plants. Ghani et al.’s review highlighted recent breakthroughs in the mediation of the silkworm humoral immune system’s response to different pathogens (Such as the RNAi pathway, which includes siRNA, miRNA and piRNA). They found that the silkworm immune system has humoral and cellular defenses against pathogens, and antimicrobial peptides (AMPs) are key components of humoral immunity. Supported by pattern recognition receptors (PRRs) and other defensive mechanisms, AMPs directly target pathogens (Ghani et al., 2025b). However, they also pointed out the redundant functions among pattern recognition receptors and signaling pathways, as well as the limitations in understanding host-pathogen interactions. Of course, Exosomal ncRNAs (such as miRNAs, lncRNAs, and circRNAs) have emerged as promising, non-invasive biomarkers for early cancer detection due to their stability, specificity, and resistance to enzymatic degradation. However, their complex roles in cellular pathways and tissue-specific expression profiles complicate functional evaluation and clinical translation. Future research must prioritize standardized protocols for exosome isolation and data normalization, comprehensive profiling of tissue-specific expression, and the integration of bioinformatics to enhance diagnostic specificity and accuracy (Ghani et al., 2025a). Similarly, ncRNAs are pivotal in vector-host-pathogen interactions. A recent review highlights that ncRNAs (e.g., lncRNA, miRNA, sfRNA, piRNA) derived from pathogens and vectors in diseases like DENV, ZIKV, and CHIKV promote pathogen proliferation and modulate host ncRNA regulation. Despite providing insights into infection stages, current research faces limitations, including the spatiotemporal specificity of ncRNA activity, insufficient characterization of functional ncRNAs, and challenges in sequencing low-abundance RNAs. Therefore, developing advanced bioinformatics tools to resolve ncRNA-target interactions is crucial (Ghani et al., 2025c).

Furthermore, ncRNA vaccines designed and developed against animal viruses are emerging as a novel therapeutic strategy. For instance, miRNAs have emerged as a significant tool in the realm of vaccinology, offering novel approaches to vaccine development. Tsetsarkin et al. developed a miRNA-targeted vaccine against tick-borne encephalitis virus (TBEV), named T/1674-mirV2, which exhibits significant immunogenicity and genetic stability in animal models (Tsetsarkin et al., 2019). Similarly, Feng et al. inserted miR-let-7b target sequences into the influenza A H1N1 genome to construct a recombinant virus named miRT-H1N1. Further studies demonstrated that miRT-H1N1 was attenuated in mice. Mice receiving intranasal vaccination with miRT-H1N1 developed robust immunity, which protected the vaccinated animals from lethal challenge with wild-type H1N1 virus (Feng et al., 2015). These findings highlight the potential of miRNA-based vaccines to induce potent immune responses. Moreover, Zhang et al. successfully developed a potent bivalent circular RNA vaccine and a sequential strategy, which provides effective protection against both individual and co-infections of PEDV and Transmissible Gastroenteritis Virus (TGEV) (Zhang W. et al., 2025). Simultaneously, a sequential regimen combining bivalent circRNA and commercial inactivated vaccines (IAV) elicited a predominantly Th1-driven antibody response, effectively neutralizing PEDV and TGEV. In the future, more ncRNA vaccines targeting animal viruses will be designed and developed.

Although many studies have demonstrated the role of ncRNAs as biomarkers in virus-host interactions and identified key molecules in the viral replication process, there are still some major challenges in the wider application of clinical medicine, especially the research and development of vaccines. For example, During the COVID-19 pandemic, mRNA vaccines targeting SARS-CoV-2 became the preferred option for human immunization (Fang et al., 2022). However, a major limitation of mRNA vaccines is their susceptibility to degradation by RNases, necessitating cold-chain storage and transportation. This poses a significant challenge for the development of animal virus vaccines. Moreover, the ability of miRNAs to bind multiple targets due to their interaction with mRNAs can potentially lead to off-target effects, affecting genes other than the one intended, resulting in unforeseen outcomes. Another noteworthy point is that the body will generate an immune response against the miRNA itself after vaccination, which may further lead to an autoimmune reaction or reduce vaccine efficacy. Of course, The development and production of ncRNA vaccines might be costly, potentially making the vaccines less accessible, particularly in regions with lower economic resources. In addition, delivery systems play a critical role in the design of ncRNA vaccines. Efficient delivery systems can ensure that the vaccine accurately reaches target cells and tissues, thereby enhancing cellular uptake and vaccination efficiency. Delivery systems such as lipid nanoparticles, viral vectors and exosomes have been proven to significantly improve the immunogenicity of ncRNA vaccines. Despite of recent advances, the current understandings in the study of the significance of ncRNAs in viral infections and antiviral immune responses are limited. For researchers, identifying targets of virus-host interactions is a great challenge, especially in the development of novel therapies and vaccines targeting these sites.

Deep learning methods based on AI are playing an increasingly important role in predicting virus-host-ncRNA interactions and are critical for decoding the molecular mechanisms underlying virus-host interactions. Zhang et al. first proposed a novel deep learning-based method (named CBIL-VHPLI) to predict virus-host protein-lncRNA interactions, and the results produced on the fine-tuned vhRPI286 dataset also demonstrated the good validity and robustness of CBIL–VHPLI in terms of predicting human lncRNA-viral protein interactions (Zhang M. et al., 2024). However, limitations remain. First, scarce binding data and inherent information bias may compromise the accurate assessment of interaction probabilities. Second, relying exclusively on sequence features leaves the investigation of these interactions incomplete.

AI algorithms have proven critical for predicting antigenic epitopes and conducting structural protein analysis, as exemplified by Google’s DeepMind AI model named AlphaFold3, which can predict the interaction between spike proteins of common viruses and antibodies, providing important insights into the mechanism of immune response and potentially leading to more effective therapies and vaccines (Desai et al., 2024). Similarly, another deep learning models, such as convolutional neural networks (CNN) and Transformers (Zhao and Wang, 2025), integrate multi-omics data to improve antigen prediction (Nasir et al., 2024), RNA secondary structure modeling (Allouche et al., 2024) and lipid nanoparticle delivery system formulation (Alshehry et al., 2025), outperforming traditional bioinformatics methods in both accuracy and efficiency. AI is presented as a useful predictive and prioritization tool, but it cannot be a substitute for experimental validation. There are still many limitations in its application to precision medicine. First, the “black-box” nature of deep learning models reduces interpretability, making it difficult to understand the basic principles behind predictions. Second, the performance of artificial intelligence models largely depends on the quality and representativeness of training data, and biased datasets may lead to prediction bias and poor generalizability (Zhao and Wang, 2025). Although developing effective inhibitors and vaccines with ncRNA as a potential target faces many challenges in the study of animal virus-host interaction, we believe a new research direction of ncRNA-based prevention and control strategies against animal viruses is on the horizon.
